# The mechanisms of ferroptosis and its role in alzheimer’s disease

**DOI:** 10.3389/fmolb.2022.965064

**Published:** 2022-08-26

**Authors:** Hongyue Ma, Yan Dong, Yanhui Chu, Yanqin Guo, Luxin Li

**Affiliations:** ^1^ Department of Neurology, Hongqi Hospital of Mudanjiang Medical University, Mudanjiang, China; ^2^ College of Life Sciences, Mudanjiang Medical University, Mudanjiang, China; ^3^ Heilongjiang Key Laboratory of Tissue Damage and Repair, Mudanjiang Medical University, Mudanjiang, China

**Keywords:** Alzheimer’s disease, ferroptosis, oxidative stress, p53, lipid peroxidation

## Abstract

Alzheimer’s disease (AD) accounts for two-thirds of all dementia cases, affecting 50 million people worldwide. Only four of the more than 100 AD drugs developed thus far have successfully improved AD symptoms. Furthermore, these improvements are only temporary, as no treatment can stop or reverse AD progression. A growing number of recent studies have demonstrated that iron-dependent programmed cell death, known as ferroptosis, contributes to AD-mediated nerve cell death. The ferroptosis pathways within nerve cells include iron homeostasis regulation, cystine/glutamate (Glu) reverse transporter (system xc^−^), glutathione (GSH)/glutathione peroxidase 4 (GPX4), and lipid peroxidation. In the regulation pathway of AD iron homeostasis, abnormal iron uptake, excretion and storage in nerve cells lead to increased intracellular free iron and Fenton reactions. Furthermore, decreased Glu transporter expression leads to Glu accumulation outside nerve cells, resulting in the inhibition of the system xc^−^ pathway. GSH depletion causes abnormalities in GPX4, leading to excessive accumulation of lipid peroxides. Alterations in these specific pathways and amino acid metabolism eventually lead to ferroptosis. This review explores the connection between AD and the ferroptosis signaling pathways and amino acid metabolism, potentially informing future AD diagnosis and treatment methodologies.

## Introduction

Programmed cell death occurs by apoptosis, necroptosis, pyroptosis, ferroptosis, and cell death associated with autophagy and unprogrammed necrosis (Moujalled et al., 2021). In 2003, Dolma et al. (2003) revealed that the compound erastin could kill tumor cells *via* an RAS oncogene mutation, but cell death was not involved in changing the nucleus and caspase-3 activation. Yang et alexpanded on these results, discovering RAS-selective lethal compound 3, later shown to be the inducer of ferroptosis (Zhang et al., 2004; Yang and Stockwell, 2008). In 2012, Dixon et alwere the first to report ferroptosis as a type of cell death (Dixon et al., 2012). The occurrence of ferroptosis is related to the metabolism of iron, amino acids, GSH, reactive oxygen species (ROS), and lipid peroxides (LPOs). Fe^3+^ in the extracellular fluid is transported to cells *via* transferrin (Tf) and subsequently reduced to Fe^2+^. Excessive H_2_O_2_ reacts with the Fe^2+^ in cells to generate a large number of ROS through Fenton reactions, which promote the generation of intracellular LPOs and trigger ferroptosis. Electron microscope examination of cellular morphology during ferroptosis showed that the membrane ruptures, bubbles develop within mitochondria, which then atrophy, the mitochondrial cristae shrink or disappear, and the membrane density increases. Furthermore, although the nuclear shape appears normal, condensed chromatin is lacking (Xia et al., 2021; Ou et al., 2022). Biological activity is also altered. ROS and iron ions aggregate, the mitogen-activated protein kinase (MAPK) system is activated, cystine uptake is reduced, GSH is depleted, and system xc^−^ is inhibited (Shin et al., 2018). A recent study found reduced iron accumulation, lipid peroxidation, and GSH and GPX4 in patients with neurodegenerative diseases. Magnetic resonance imaging showed that iron deposition is correlated with cognitive impairment, and this deposition is mainly observed in the hippocampus, cortex, and basal ganglia, where brain cells experience oxidative stress, lipid peroxidation, and increased cystine/Glu transporter expression, iron metabolism, and balance (Ghadery et al., 2015; Masaldan et al., 2019). Iron is deposited in the brain cells of AD patients, and excessive iron will exacerbate oxidative damage and cognitive deficits (Bao et al., 2021). Given this evidence an in-depth understanding of ferroptosis mechanisms involved in the occurrence and development of AD is needed to facilitate timely diagnosis and treatment before major brain damage occurs, improving the survival rate and quality of life of AD patients. The purpose of the present review is to summarize the mechanism of ferroptosis in nerve cells and further analyze the possible pathway of ferroptosis involved in AD, summarize the relationship between ferroptosis-related drugs and AD, and propose methods for future clinical practice.

## Mechanisms of ferroptosis

### Iron homeostasis and ferroptosis

The maintenance of iron homeostasis is essential for normal physiological function. There are two forms of iron in cells: Fe^3+^ and Fe^2+^. As a storage and transportation form of iron, Fe^3+^ is relatively stable. Fe^2+^ can transfer electrons, participate in various oxidation-reduction reactions and act as a reaction catalyst. An imbalance in iron homeostasis can result in lipid peroxidation and cellular oxidative stress, ultimately leading to ferroptosis. Therefore, the transfer in, transfer out, storage, and turnover of iron play important roles in the ferroptosis process.

Iron is transported by cells in two forms, Tf bound and non-Tf-bound iron. Tf is the primary protein responsible for iron transport. Under physiological conditions, Fe^3+^ is transferred into brain microvascular endothelial cells through endocytosis mediated by transferrin receptor 1 (TfR1) and Tf on the luminal side of the cells (Masaldan et al., 2019). In the acidic environment of the endosome, Fe^3+^ is reduced to Fe^2+^ by the six-transmembrane epithelial antigen of prostate 3 (Derry et al., 2020; Li et al., 2020; Reichert et al., 2020; Jia et al., 2021). The divalent metal transporter 1 or zinc-iron regulatory protein family 8/14 can assist in moving the iron into the labile iron pool (LIP) (Derry et al., 2020; Li et al., 2020; Reichert et al., 2020; Jia et al., 2021; Qu et al., 2022). Under normal physiological conditions, oxidation–reduction activity of Fe^2+^ in the form of LIP is maintained at a low concentration (approximately 0.2–0.5 µM) to meet metabolic requirements (Petrat et al., 1999).

GSH has a high affinity with Fe^2+^ and the major component of LIP in the cytosol is presented as the GSH-Fe^2+^ conjugates (Lv and Shang, 2018). A decrease in intracellular GSH , increases the concentration of Fe^2+^ facilitating the Fenton reaction. The storage of labile iron in ferritin serves to circumvent its high reactivity, avoiding the generation of reactive species (Reichert et al., 2020). Ferritin is an intracellular complex of 24 subunits (composed of heavy and light ferritin chains) that stores up to 4,500 iron atoms in an inactive oxidized and reduced forms to protect cells and tissues from oxidative damage (De Domenico et al., 2009). The primary function of ferritin is to maintain the equilibrium between the reduced (Fe^2+^) and oxidized states (Fe^3+^) (Reichert et al., 2020). Ferritin can catalyze excessive intracellular Fe^2+^ into non-toxic Fe^3+^ in the presence of proteins related to iron metabolism, which are closely bound and stored in the ferritin complex to maintain iron homeostasis (Reichert et al., 2020). An iron metabolism imbalance—caused by abnormal ferritin—will induce ferroptosis (Mancias et al., 2015; Tang et al., 2018). Nuclear receptor coactivator 4 (NCOA4) is a selective carrier receptor that can perform selective autophagy of ferritin (ferritinophagy) when intracellular iron levels are low so that iron is released (Ling et al., 2017). Arginine on the surface of the heavy ferritin chain FTH1 binds to a C-terminal domain of NCOA4 when iron levels are low, thereby promoting the transfer of autophagosomes to lysosomes (Mancias et al., 2015; Tang et al., 2018; Cheng et al., 2021). Mancias et al. (2015). demonstrated that the amount of NCOA4 depends on whether it interacts with the HERC2 protein. NCOA4 on the autophagosome targets the HERC2 protein when intracellular iron levels are high and is degraded by the proteasome. NCOA4 degradation ultimately reduces the breakdown of ferritin. NCOA4-mediated ferritinophagy participates in some physiological processes associated with iron metabolism in the cell, including erythropoiesis. Recent evidence suggests that autophagy is a conserved catabolic cellular pathway. Moreover, the loss of HERC2 causes severe neurodevelopmental abnormalities, contributing to neurogenetic diseases (Morice-Picard et al., 2016; Tang et al., 2018). Therefore, it is thought that HERC2 deficiency would result in a malfunctioning response to elevated iron levels, resulting in ferritinophagy, free iron release, and neuronal cell damage.

Studies have shown that the intracellular LIP level is regulated by ferritin (Reichert et al., 2020), and in ferroptosis, ferritinophagy increases LIP (Dixon and Stockwell, 2014), which can activate the Fenton and Haber–Weiss reactions to generate ROS (Lane et al., 2018). Specifically, the ROS H_2_O_2_ oxidizes Fe^2+^ to Fe^3+^ *via* the Fenton reaction, forming the highly active hydroxyl radical (·OH), inducing oxidative stress and leading to ferroptosis, the accumulation of lipoid-OOH, and the oxidation of polyunsaturated fatty acids (PUFAs) (Kehrer, 2000; Qi et al., 2020).

To date, ferroportin 1 (FPN1, also known as solute carrier family 40 member 1) is the only nonheme cellular iron exporter identified in mammals. It transports iron from iron storage cells into the blood to optimize systemic iron homeostasis. In the central nervous system, FPN1 is distributed in most cell types, including neurons, astrocytes, oligodendrocytes, and brain microvascular endothelial cells (Bao et al., 2021).In neurons, amyloid precursor protein (APP) connects to FPN1 and stabilizes the expression level of FPN1. APP is also an iron oxidase that can oxidize Fe^2+^ to Fe^3+^ and transfer it out of cells (Duce et al., 2010). FPN1 is essential to embryonic development: mice with a global FPN1 deletion are embryonically lethal (Drakesmith et al., 2015).

### Cystine/glutamate reverse transport system, GPX4 and ferroptosis

System xc^−^ is a heterodimer composed of two solute carriers, solute carrier family 3A2 (SLC3A2) and solute carrier family 7A11 (SLC7A11). Through system xc^−^, Glu and cystine enter and leave the cell in equal amounts. Cystine, which is ingested, is then reduced to gamma-glutamylcysteine (γ-Glu-Cys) in the cell, which is involved in the synthesis of GSH. The excitatory amino acid Glu can induce the death of nerve cells, which is iron-dependent. It is speculated that Glu-induced death and ferroptosis may share the same signaling pathway. Studies have shown that system xc^−^ is inhibited by ferroptosis (Sato et al., 2018; Li et al., 2020). Specifically, system xc^−^ is inhibited by high extracellular Glu concentration, and decreasing intracellular GSH leads to peroxidase 4 (GPX4) inhibition and lipoxygenase (LOX) activation. Eventually, lipid peroxidation and cellular oxidative stress are generated, resulting in cell ferroptosis (Huang et al., 2020).

p53 plays a role in regulating ferroptosis *via* SLC7A11. The p53 tumor suppressor is “the guardian of the genome” that participates in the control of cell survival and division under various stresses. Beyond its effects on apoptosis, autophagy, and the cell cycle, p53 also regulates ferroptosis through a transcriptional or posttranslational mechanism. p53 can enhance ferroptosis by inhibiting the expression of SLC7A11 (Kang et al., 2019). A complete transactivation domain is necessary for p53 to regulate SLC7A11 and ferroptosis. Although mutant p53 inhibits the expression of SLC7A11, thereby promoting ferroptosis (Jiang et al., 2015a; Latunde-Dada, 2017). p53 mutants do not inhibit SLC7A11 (Jiang et al., 2015b; Liu and Gu, 2022). Recent reports indicate a more complex mechanism in which wild-type p53 may enhance survival advantage by promoting antioxidation in some cases, and p53-mediated activation of p21 (encoded by CDKN1a) inhibits phospholipid oxidation by protecting intracellular mercaptans (including GSH) (Tarangelo et al., 2018; Hu et al., 2020; Tang et al., 2021). In addition, p53 plays a crucial role in regulating dynamic ROS. Jiang et al. found that p53 regulates antioxidant response under short-term stress, assisting cell recovery. However, continued activation of p53 triggers a pro-oxidation reaction that induces cell death. An ROS increase in the late stage of p53 activation is partly due to the inhibition of SLC7A11 (Jiang et al., 2015b). Chu et al. showed that p53 could indirectly trigger arachidonate 12-lipoxygenase (ALOX12) function through transcriptional inhibition of SLC7A11, thus leading to ALOX12-dependent ferroptosis resulting from ROS stress (Chu et al., 2019).

Cells have several death escape mechanisms. In the ferroptotic process, one of the most important and most studied mechanisms involves the enzyme glutathione peroxidase 4 (GPX4) (Reichert et al., 2020). In mammals, the GPX family consists of eight members. GPX1–GPX4 are selenoproteins that contain selenocysteine in the catalytic center. GPX4 is the only enzyme known to reduce complex phospholipid hydroperoxides directly (Xia et al., 2021). Hydroperoxides can activate catalytic reactions in the presence of transition metals such as iron, which eventually leads to ferroptosis. Therefore, GPX4 is key to cell survival (Friedmann Angeli et al., 2019). GSH is part of an intracellular antioxidant system that plays an important role in free radical scavenging, anti-aging and antioxidation activities, and other major physiological functions. In ferroptosis, GPX4 uses GSH as a substrate to mediate the lipid-OOH conversion to lipid-OH, and the sulfhydryl group in GSH reduction is readily dehydrogenated to form oxidized glutathione disulfide (GSSG), which plays an antioxidant role. GSSG is reduced to GSH by GSH reductase in NADPH-participating reactions (Qiu et al., 2020; Lei et al., 2021). GSH is continuously produced by glutamate cysteine ligase (GCL) and glutathione synthetase (GSS), and GCL activity is the rate-limiting step in GSH synthesis (Thompson et al., 2009; Parpura et al., 2017; Stockwell et al., 2017; Abdalkader et al., 2018; Shi et al., 2021; Yan et al., 2021). When reduced GCL and GSS activity limits the synthesis of GSH, GPX4 is eventually inactivated, resulting in the accumulation of lipid peroxidation, further ROS production, and ultimately, ferroptosis.

### Lipid metabolism and ferroptosis

Initiation of lipid peroxidation requires the removal of a bis-allylic hydrogen atom (located between two carbon–carbon double bonds) from polyunsaturated fatty acyl moieties in phospholipids (PUFA-PLs) incorporated into lipid bilayers. This process leads to the formation of a carbon-centered phospholipid radical (PL•) and subsequent reaction with molecular oxygen to yield a phospholipid peroxyl radical (PLOO•), which removes hydrogen from another PUFA, forming PLOOH. If not converted to the corresponding alcohol (PLOH) by GPX4, PLOOH and lipid free radicals—in particular, PLOO• and alkoxyl phospholipid radicals (PLO•)—will react with PUFA-PLs to propagate PLOOH production by removing more hydrogen atoms and reacting with molecular oxygen. This reaction eventually leads to the formation of lipid peroxide breakdown products. A consequence of this chain reaction is the eventual deterioration of membrane integrity and, ultimately, destabilization of organelles or cell membranes. Therefore, membranes with a high PUFA-PL content would be especially susceptible to peroxidation, as has been shown to occur in neurons (Jiang et al., 2021). Acyl-CoA synthetase long-chain family member 4 (ACSL4) determines a cell’s sensitivity to ferroptosis (Shin et al., 2018). Responsible for the esterification of PUFA into acyl-COA, ACSL4 promotes PUFA fatty acid activation. Activated the fatty acids under the action of Lysophosphatidylcholine Acyltransferase 3 (LPCAT3) transferred to inside and outside the cell membrane and esterification; the substrates can undergo peroxidation resulting in the formation of arachidonoyl (AA) and adrenoyl (AdA) acids. During this process, many LPOs and lipid ROS are formed, aggravating oxidative stress and contributing to ferroptosis (Friedmann Angeli et al., 2019; Reichert et al., 2020; Jia et al., 2021).

**FIGURE 1:**
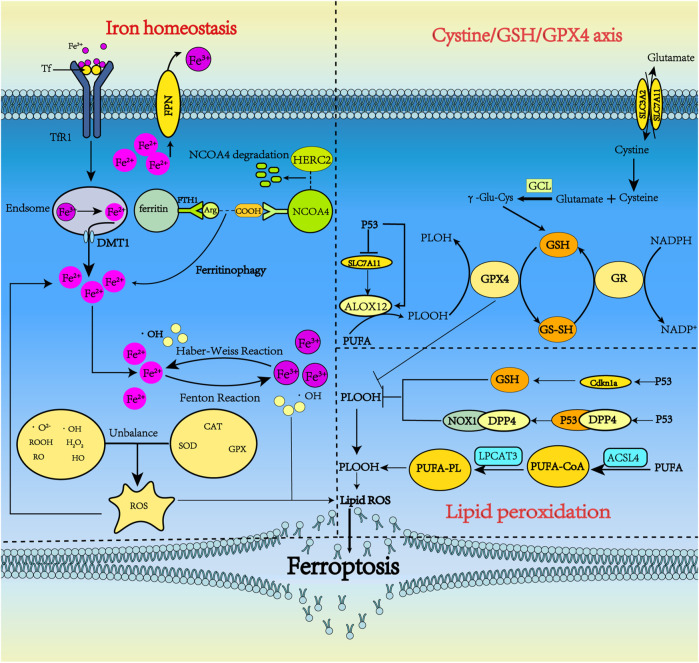
The metabolism pathways for ferroptosis. Ferroptosis can occur through three major pathways, iron homeostasis, the cystine/GSH/GPX4 axis, and lipid peroxidation. 1) Iron homeostasis. Tf carrying Fe^3+^ forms a complex with TfR1 and enters neurons via clathrin-mediated endocytosis. Fe^3+^ detaches from Tf and then is reduced by STEAP3. Fe^2+^ is pumped into the cytoplasm by DMT1 and is stored in ferritin in the form of Fe^3+^ when overloaded. Under some conditions, ferritin undergoes autophagy by binding with NCOA4, releasing iron, which subsequently leads to lethal iron levels and ferroptosis. NCOA4 can interact with HERC2, leading to NCOA4 degradation. Ferritinophagy increases LIP, which can activate the Fenton and Haber-Weiss reactions to generate ROS. 2) Cystine/GSH/GPX4 axis. System xc^−^ includes two chains: a specific light chain, SLC7A11, and a heavy chain, SLC3A2. Through system xc^−^, Glu and cystine enter and leave the cell in equal amounts. Cystine, which is ingested, is then reduced to γ-Glu-Cys in the cell and becomes involved in the synthesis of GSH. GSH is continuously produced by GCL and GSS. In ferroptosis, GPX4 uses GSH as a substrate to mediate the lipid-OOH conversion to lipid-OH, and the sulfhydryl group in GSH reduction is readily dehydrogenated to form oxidized glutathione disulfide (GSSG), which plays an antioxidant role. P53 could indirectly trigger arachidonate 12-lipoxygenase (ALOX12) function through transcriptional inhibition of SLC7A11, thus leading to ALOX12-dependent ferroptosis resulting from ROS stress. 3) Lipid peroxidation. PUFA produces a large amount of lipid ROS through the continuous action of ACSl4 and LPCAT3. p53 can inhibit ferroptosis by inhibiting DPP4 activity or inducing CDKN1A expression.

p53 also plays a role in lipid peroxidation. Cell membrane PUFAs can undergo peroxidation reactions *via* LOXs, iron-containing enzymes that also induce cell ferroptosis. p53 activates the LOX enzyme ALOX12 to induce ferroptosis in cells independent of GPX4 activity (Chu et al., 2019; Li and Li, 2020). Inhibition or knockdown of ALOX12 may be a new approach to interrupt ferroptosis (Ou et al., 2016). In contrast, loss of p53 prevents the accumulation of dipeptidyl-peptidase-4 (DPP4) in the nucleus, triggering membrane-associated DPP4-mediated lipid peroxidation. This peroxidation promotes the interaction between DPP4 and nicotinamide adenine dinucleotide phosphate oxidase 1 (NOX1), resulting in the formation of the NOX1–DPP4 complex, which mediates plasma membrane lipid peroxidation and ferroptosis (Xie et al., 2017; Li and Li, 2020). p53 inhibits ferroptosis by reducing the accumulation of toxic lipid ROS and inducing the expression of cyclin-dependent kinase inhibitor 1A (CDKN1A/p21) (Figure 1) (Li and Li, 2020; Song and Long, 2020).

### Glutamate-storage, uptake and recycling

Glu, glutamine (Gln) and cysteine play vital roles in ferroptosis. The GSH molecule consists of glutamic acid, cysteine, and glycine (Gly), with cysteine being the limiting substrate in its formation. GSH plays an important role in cells and is the main low molecular weight antioxidant, regulating various important functions. Furthermore, Glu is not only one of the major excitatory amino acids in the brain, but also participates in the Glu–Gln cycle, which links glucose and amino acid metabolism to synaptic transmission, cellular homeostasis, and cellular energy metabolism (Figure 1). Therefore, Glu storage, synthesis, receptor signaling and transport, uptake and recycling are closely related to brain energy metabolism.

**FIGURE 2:**
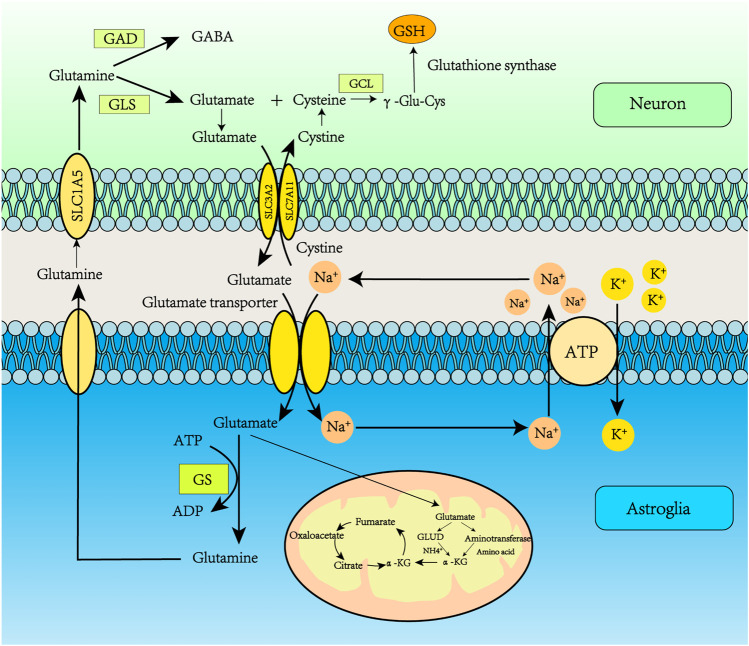
Glutamate recycling via the glutamate-glutamine cycle. Glu transporters are mainly distributed in astrocyte synapses. Glu binds to transporters which deliver it to the astrocyte cytoplasm. Glu transporters are co-transported into astrocytes by Na^+^ and Glu, and Na^+^ is transported to the extracellular space by Na^+^/K^+^-ATPase. Glu reacts with GS to produce Gln, and the ATP consumed in this process may be supplied by glycolysis. The resulting Gln is released into the neuron, and then Gln enters the neuron through the SLC1A5 receptor. The Gln absorbed by the neuron is converted into Glu under the action of glutaminase. GABA can also be produced by the action of Glu decarboxylase. In addition, Glu can be converted to α-ketoglutarate by Glu dehydrogenase or aminotransferase, participating in the tricarboxylic acid (TCA) cycle, which provides citrate and oxaloacetate for lipid synthesis and converts Glu to aspartate.

Glu transporters are mainly distributed in astrocyte synapses. The Glu bind to transporters which move it to the astrocyte cytoplasm. Glu transporters are co-transported into astrocytes by Na^+^ and Glu, and Na^+^ is transported to the extracellular space by Na^+^-K^+^-ATPase. Glu reacts with Gln synthetase (GS) to produce Gln; the ATP consumed in this process may be supplied by glycolysis, in which GS is only expressed in astrocytes (Parpura et al., 2017). Therefore, the Glu/Gln cycle plays a key role in maintaining Glu levels in the central nervous system (Andersen et al., 2021). The resulting Gln is released into the neuron, entering through the SLC1A5 receptor. The Gln absorbed by the neuron is converted into Glu under the action of glutaminase. GABA can also be produced by the action of Glu decarboxylase. In addition, Glu can be converted to α-ketoglutarate by Glu dehydrogenase or aminotransferase, then participate in the tricarboxylic acid (TCA) cycle, which provides citrate and oxaloacetate for lipid synthesis and converts Glu to aspartate (Haddad et al., 2021). Glu can be introduced into the TCA cycle when glucose supply is limited. p53 promotes Glu decomposition by up-regulating the expression of glutaminase 2 (Figure 2) (Liu and Gu, 2021).

Glu receptors are roughly divided into ionic and metabolic types. Ionic Glu receptors include those of N-methyl-D-aspartate (NMDA), among the most important postsynaptic Glu receptors. Mediating the flow of Ca^2+^ (Liu et al., 2021), the NMDA receptor is a heterotetramer calcium channel, mainly composed of two NR1 subunits and two NR2 subunits (Furukawa et al., 2005). In the hippocampus, the NR2 subunit is mainly expressed as NR2A and NR2B. The synaptic NMDA receptors rich in NR2A subunits are primarily activated by the cAMP response element-binding protein, and brain-derived neurotrophic factor (BDNF) gene expression induces cell survival events (Leveille et al., 2008). BDNF is an important neurotrophic factor expressed in many brain regions such as the hypothalamus, cortex, brainstem and hippocampus. It plays a key role in the survival, differentiation, and growth of neuronal dendrites and axons, and the regulation of synaptic plasticity. Vesicular Glu transporters (VGLUT1) are located in the glutamatergic presynaptic vesicle plasma membrane, controlling Glu transport into the synaptic vesicle. The VGLUT1 quantity and the extracellular Glu concentration determine the speed and efficiency of transport (Chen et al., 2011).

The tripeptide GSH is the most abundant endogenous antioxidant in the body, removing free radicals and maintaining balance in the oxidative defense system. Several studies have shown that the first step in GSH synthesis involves the formation of γ-Glu-Cys in an ATP-dependent reaction catalyzed by GCL, which requires Mg^2+^ or Mn^2+^ as cofactors (Liu et al., 2021). γ-Glu-Cys can significantly increase GSH and the GSH/GSSG ratio (Liu et al., 2021). This step is rate-limiting because it depends on cysteine bioavailability and GCL activity. In the second step, γ-Glu-Cys and Gly form GSH *via* GSS activity (Stockwell et al., 2017). The cell’s ability to biosynthesize GSH is controlled by various factors, including intracellular substrate utilization (L-cysteine), GCL activity, rate-limiting enzymes in GSH synthesis, and GSH feedback inhibition of GCL (Haddad et al., 2021).

The most significant difference between ferroptosis and other types of programmed cell death is the change in mitochondrial morphology (Luo et al., 2021). Ferroptotic mitochondria are smaller with increased membrane density (Bao et al., 2021) and elevated cytoplasmic and lipid ROS radicals (Dixon et al., 2012; Johnson et al., 2021). ROS are partially reduced oxygen-containing molecules, including superoxide (O_2_•^−^), peroxides (H_2_O_2_ and ROOH), and free radicals (HO• and RO•) (Latunde-Dada, 2017; Chen et al., 2020; Foret et al., 2020). Superoxide is the most important free radical (Serviddio et al., 2015). Excessive ROS can be detoxified by antioxidants (enzymes and non-enzymes) and in reactions catalyzed by superoxide dismutase (Cu-SOD, Zn-SOD, and Mn-SOD), GPX and catalase. ROS are produced by glucose and glutamine (Gln) metabolism, which reduces GSH and GPX4 levels (Chen et al., 2020). An imbalance in ROS production and detoxification rates leads to oxidative stress, and the subsequent radicals generate damage DNA, proteins, and lipids (Chen et al., 2020). Under oxidative stress, high levels of superoxide can induce compounds including iron (4Fe-4S) clusters, heme, and ferritin to release Fe^2+^, which causes ferroptosis through Fenton and Haber–Weiss reactions. In ferroptosis, SLC7A11 and GSH depletion lead to iron-dependent ROS accumulation (Dixon and Stockwell, 2014).

## Ferroptosis and alzheimer’s disease

### Iron homeostasis and alzheimer’s disease

Regulation of iron homeostasis is important for maintaining normal brain function, and dysregulation of iron homeostasis in the brain can lead to oxidative stress and inflammatory responses, resulting in cell damage and ultimately neurodegenerative diseases. Typical neuropathological features of AD include the deposition of beta-amyloid (Aβ) into neuroinflammatory plaques, intracellular aggregates of Tau protein in neurofibrillary tangles, synaptic loss, neuroinflammation, and neuronal death. In addition to these typical pathologies, MRI data of AD patients have shown iron deposition in the hippocampus, cortex, and basal ganglia (Ghadery et al., 2015; Masaldan et al., 2019). Subsequent studies revealed increased iron, Tf, and ferritin in the brain (Ashraf et al., 2020; Bao et al., 2021). These findings suggest that neuronal cells in AD disease upregulate ferritin and downregulate FPN expression, increasing iron intake and reducing iron excretion. This process leads to increased free Fe^2+^ in cells and increased ferritin, manifested by iron deposition in the brain.

In AD, cytotoxicity induced by Aβ1-42 directly induces down-regulation of FPN in primary neurons and the hippocampus. Abnormal phosphorylation of the tau protein can lead to increased APP (Bao et al., 2021), a precursor of Aβ production and aggregation (Derry et al., 2020). APP is first broken down by either α-secretase or β-secretase and then by γ-secretase. In the physiological state, α-secretase is the first to cleave APP for the non-amyloidosis pathway. However, if APP is first cleaved by β-secretase, neurotoxic Aβ is produced (Tsatsanis et al., 2020). The protein furin plays a critical role in regulating the rate of proteolytic activation of α-secretase and β-secretase. Furin concentration is positively correlated with α-secretase activity but negatively correlated with β-secretase activity. Iron deposition results in reduced furin transcription and translation, thereby enhancing β-secretase activity by reducing furin protein expression. Enhanced β-secretase activity increases Aβ production through the amyloidosis pathway (Ward et al., 2014). The damaging cycle then continues with Aβ-induced downregulation of FPN and iron accumulation. Generally, the tau protein can mediate APP’s interaction with FPN on the cell surface to promote iron excretion. However, reduced tau protein is associated with AD, affecting FPN’s ability to excrete iron (Wang and Mandelkow, 2016; Ayton et al., 2020; Derry et al., 2020). Furthermore, increased FTH levels in AD are associated with lower FPN levels (Ashraf et al., 2020). Everett et al. found that amyloid plaques reduced Fe^3+^ to Fe^2+^ (Everett et al., 2014a), and Aβ could transform Fe^3+^ stored as ferrihydrite into redox-active biological substances containing Fe^2+^ (Everett et al., 2014b; Derry et al., 2020).

Due to increased iron intake and decreased iron excretion, increased intracellular liberation of Fe^2+^ activates the ferroptosis pathway. First, excessive Fe^2+^ enhances the Fenton reaction and produces oxhydryl radicals. Second, it promotes lipid peroxide production, which ultimately triggers ferroptosis.

### GPX4 and alzheimer’s disease

GPX4, an antioxidant enzyme, is highly expressed in NCOA4 deficient mice (Bellelli et al., 2016). Yoo et alfound that GPX4 deletion in adult mice leads to mitochondrial damage, neurodegeneration in the hippocampus, and astrocyte proliferation (Yoo et al., 2012). Moreover, Hambright et al. (2017) found significant neurological deficits and cognitive impairment in GPX4-deficient mice. When Bao et al. (2021) injected Aβ into the brain of mice, they found elevated levels of iron and ferritin in the hippocampus and decreased levels of GPX4, suggesting that Aβ directly affects ferroptosis in neurons. Thus, GPX4 inhibition in ferroptosis offers protection against neurodegeneration (Cardoso et al., 2017). As a substrate of GPX4, GSH plays an antioxidant role. Studies have shown that γ-Glu-Cys can significantly increase GSH, increase the GSH/GSSG ratio, and decrease the generation of Aβ and oxidative stress (Liu et al., 2021). In AD patients, the GSH content is reduced (Ashraf et al., 2020), so effectively preventing GSH decrease is a promising new treatment strategy for AD occurrence and development.

### Lipid metabolism and alzheimer’s disease

Recent studies have shown that lipid ROS in ferroptosis may cause AD. Although the highest PUFA content is found in adipose tissue, PUFA in brain tissue accounts for 30%–35% of the total fatty acid content, so the central nervous system is very vulnerable to lipid peroxidation (Peng et al., 2021). ACSL4 plays an important role in PUFA activation and determines ferroptosis sensitivity (Shin et al., 2018). Yan et al. (2022) analyzed the hippocampal transcriptome of the APP/PS1 mouse model and found elevated expression of ACSL4. Unfortunately, it is not known whether ACSL4 expression or activity is modified by AβO.

Dietary arachidonic acid (ARA) is the second most common type of PUFA in meninges phospholipids, where lipid peroxidation readily occurs, leading to lipid bilayer damage. Recent research shows that increased ARA intake induces cognitive alteration and increases the neurotoxicity of amyloid-β peptide (Aβ) (Thomas et al., 2017), which leads to AD. In addition to its involvement in synaptic plasticity and transmission, free ARA plays a crucial role in neuroinflammation through its conversion into various eicosanoids by cyclooxygenases, prostaglandin synthases, and lipoxygenases, the activities of which have been associated with neurodegenerative diseases (Czapski et al., 2016). As the brain’s consumption and metabolism of ARA are up-regulated in AD patients, suggesting that ARA is involved in the pathomechanism of this disease (Esposito et al., 2008), ARA consumption could constitute a risk factor for AD in humans and should be considered in future preventive strategies. ARA is specifically released from membrane phospholipids by cytosolic phospholipase A2 (cPLA2), which is translocated to the membranes in a cytosolic calciumdependent manner after its phosphorylation on Ser505 by MAPK. cPLA2 is activated by Aβoligomers. Its pharmacological inhibition or the suppression of its expression protects neuronal cells against the neurotoxicity of Aβ oligomers and preserves cognitive abilities (Czapski et al., 2016). The ARA released *via* cPLA2 induction can be metabolized by COX or LOXs (Chuang et al., 2015). Among LOX alterations, those of LOX12/15 can lead to oxidative stress, resulting in free radical-dependent DNA damage and poly (ADP-ribose) polymerase-1 overactivation, neuronal degeneration and death (Czapski et al., 2013). Therefore, treating AD with LOX has broad prospects.

### Amino acid metabolism and alzheimer’s disease

Amino acids play an important role in the occurrence and development of AD. Glu is an important excitatory neurotransmitter in the body, helping to transmit information between nerve cells. During post-translational modification, many proteins undergo glycation reactions between their free reducing sugars and free amino groups. Some studies have shown increased levels of advanced glycation end-products (AGEs) in the brains of AD patients, suggesting that AGEs play an important role in activating microglia and Aβ deposition in AD (Byun et al., 2012). AGEs are irreversible adducts of the Maillard reaction that accumulate in the brain as we age. Glyoxal or methylglyoxal (MG) can contribute to AGE production (Currais and Maher, 2013). MG is primarily removed *via* the glyoxalase system, composed of Glo-1 and Glo-2. Glo-1 is the rate-limiting enzyme for the system and is dependent on GSH (Bijnen et al., 2018). Glo-1 activity also depends on the cellular redox state and the GSH/GSSG ratio (Haddad et al., 2021). AGE binding to albumin secreted by microglia results in toxicity and subsequent Aβ aggregation. Aβ can promote the release of Glu from vesicles into the synaptic cleft, leading to the activation of extrasynaptic N-methyl-D-aspartate receptors (NMDARs). Over-activation of NMDARs leads to calcium overload of postsynaptic neurons, inducing excitatory toxicity, neuronal apoptosis, and neurodegeneration. Kashani et al. (2008). found decreased Glu transporter expression in the cerebral cortex in patients with AD. VGLUT1is significantly decreased, possibly resulting in poor clearance of glutamic acid in the synaptic cleft, leading to excitatory toxicity. Furthermore, high extracellular Glu will inhibit system xc^−^, leading to ferroptosis.

The presence of amyloidoligomers (AβO) is closely correlated to the incidence of AD. Soluble AβO is currently considered the main source of brain neuron injury and central nervous system degeneration (Reiss et al., 2018). As an intermediate product of Aβ fibrosis, AβO is significantly more toxic than monomers and fibers. AβO can be classified as low molecular weight (<50 kDa) and high molecular weight (>50 kDa), and different sizes and morphologies of oligomers may produce different pathological effects. For example, in a mouse model, low molecular weight AβO (e.g., dimer and trimer) can significantly inhibit the long-term enhancement of hippocampal neurons and damage the spatial memory function. In contrast, high molecular weight soluble Aβ aggregates are more likely to induce microglial activation, resulting in neuroinflammatory responses (Figueiredo et al., 2013). AβO can interact with metabolic Glu receptor 5 to promote long-term depression and inhibit long-term potentiation in the hippocampus, leading to downstream responses and kinases activation. For example, p38-MAPK, the end of Jun N-terminal kinase (JNK), and cell-cycle dependent kinase affect the plasticity of gene transcription (Ittner et al., 2010). Furthermore, p38MAPK and JNK have been associated with AD-like lesions caused by diabetes mellitus (Kim and Song, 2020). Hyperphosphorylation of the tau protein can be mediated by activation of the p38MAPK/p53 signaling pathway (Sun et al., 2017). In addition, increased p53 expression inhibits the expression of the system xc^−^ SLC7A11, resulting in reduced uptake of cystine, decreased GSH peroxidase activity, reduced cell antioxidant capacity, and increased sensitivity of cells to ferroptosis (Kang et al., 2019).

**FIGURE 3:**
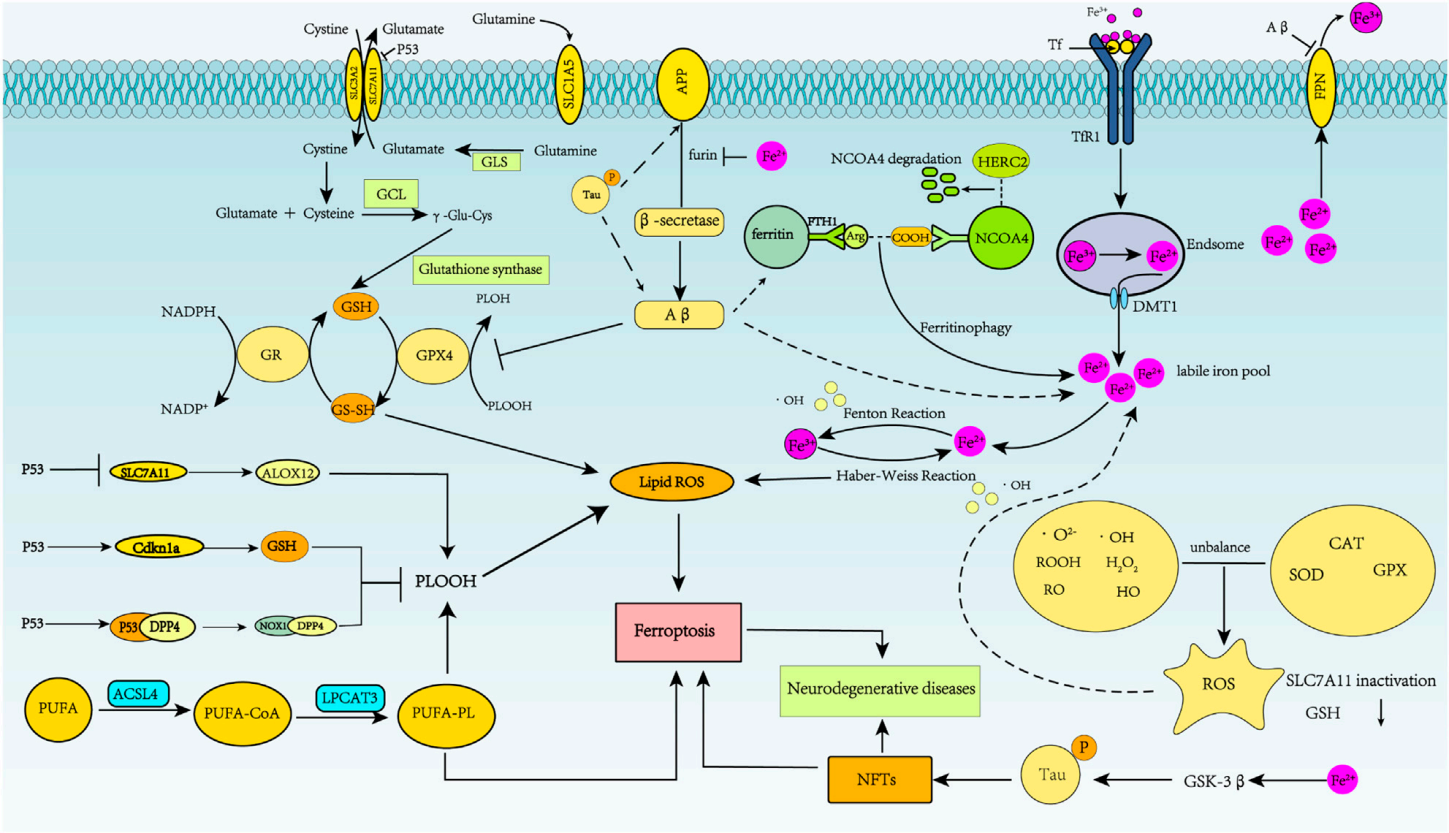
Schematic representation of ferroptosis regulation of Alzheimer’s disease. In AD, APP is first cleaved by β-secretase, and neurotoxic Aβ is produced. Abnormal phosphorylation of the tau protein can lead to increased APP and Aβ40 aggregation. Fe^2+^ enhances β-secretase activity by reducing furin protein expression, thereby increasing Aβ production through the amyloidosis pathway. Aβ1-42 directly induces down-regulation of FPN. Increased intracellular liberation of Fe^2+^ activates the ferroptosis pathway. Aβ can decrease levels of GPX4 and elevate levels of ferritin. Excessive iron in neurons can lead to tau hyperphosphorylation and NFT formation through the GSK-3β kinase pathway.

Neuropathological AD changes have been associated with impaired cerebral insulin signaling (Takeda et al., 2010; Martinez-Valbuena et al., 2019), and decreased insulin signaling in the brain can inhibit phosphatidylinositol 3-kinase/Akt and activate GSK-3β (Ma et al., 2015). Excessive iron in neurons can lead to tau hyperphosphorylation and NFT formation through the CDK5/P25 complex and GSK-3β kinase pathway (Yan and Zhang, 2019). GSK-3β phosphorylates Nrf2, leading to Nrf2 degradation (Chen et al., 2020; Qu et al., 2020). Kanninen et al. reviewed the neuroprotective role of Nrf2 in AD, with special emphasis on the role of GSK-3β in the Nrf2 pathway (Kanninen et al., 2011). Moreover, it was reported that GSK-3β inhibition in SAMP8 mice results in increased nuclear Nrf2 and total GST in the cortex (Farr et al., 2014; Qu et al., 2020). The complex roles played by ferroptosis in AD regulation are shown in Figure 3.

### Ferroptosis inhibitors and clinical application

Ferroptosis inhibitors eliminate free radicals, inhibiting enzymes that produce lipids or LPOs and reducing free iron. Iron inhibitors are classified as aromatic amine antioxidants, α-tocopherol, nitroxides, natural polyphenol compounds, ACSL4 inhibitors, LOX inhibitors, or other types (Table 1). As patients with AD have iron deposits in their brain cells, and excess iron can exacerbate oxidative damage and cognitive deficits, ferroptosis inhibitors offer broad prospects for treating AD.

**TABLE 1:** Summary of the available ferroptosis Inhibitors in Alzheimer disease.

Sort	Inhibitors	Mechanism of action	Experimental models	Effector cell	References	
Aromatic amine antioxidants	Ferrostatin-1	Block ROS production and lipid peroxidation	Aβ induced C57 mice	Primary hippocampal neurons	Bao et al. (2021)	Moreau et al. (2018); Feng et al. (2020); Mao et al. (2020); Xu et al. (2021)
	liproxstatin-1	Inhibit lipid peroxidation and up-regulate GPX4 expression	Aβ induced C57 mice	Primary hippocampal neurons	Li et al. (2019); Bao et al. (2021)	
α-tocopherol	Vitamin E	Inhibit lipid peroxidation and maintain the integrity of cell membrane	Ttpa^−/−^mice, Ttpa^−/−^APPsw mice	Cerebellum cortex hippocampus Purkinje neurons	Boccardi et al. (2016); Gugliandolo et al. (2017); Kryscio et al. (2017)	
Nitroxides		Participate in Fenton reaction, inhibit the production of hydroxyl radical	—	—	Shi et al. (2017)	
Natural polyphenol compounds	Baicalein	Inhibits GSH depletion, GPX4 degradation and lipid peroxidation, increases Nrf2, and inhibits 12/15-LOX	APP/PS1mice, C57/BL6 mice, HT22 cells	Hippocampus	Xie et al. (2016); Li et al. (2019); Yuan et al. (2020)	
	Curcumin	Chelates iron, reduces iron accumulatio, inhibits Aβ aggregation, and reduces the effects of P-tau protein	SH-SY5Ycells, APP/PS1mice,5×-familial AD (5XFAD)	Hippocampal CA1 area	Tang and Taghibiglou, (2017); Reddy et al. (2018); Ege, (2021)	
	EGCG	Antioxidant anti-inflammatory and neuroprotective effects; reduces Aβ production	APP/PS1 mice	Primary cortical neurons	Cascella et al. (2017); Plascencia-Villa and Perry, (2021)	
	Melatonin	Reduce oxidative stress and stimulate the synthesis of antioxidant enzymes (SOD, GPX, and glutathione reductase) and GSH production	APP/PS1 mice, N2a/APP cells, APP 695 transgenic mice	Neuron (mitotrondria endoplasmic reticulum)	Balmik and Chinnathambi, (2018)	
	Gingko biloba	Inhibit lipid peroxidation	Wistar rats	Hippocampus, striatum and substantia nigra	Bridi et al. (2001); Plascencia-Villa and Perry, (2021)	
	CMS121	Regulates lipid metabolism, reduces inflammation and lipid peroxidation	APPswe/PS1ΔE9 transgenic mice, HT22 neuronal cell, BV2 microglial cells, C65 cells	Hippocampus	Ates et al. (2020)	
LOX inhibitors	Zileuton	Decreased γ-secretase, Aβ and Tau	3xTg mice model	—	Di Meco et al. (2014)	
Other inhibitors	Vitamin C	Promote the production of endogenous antioxidants (GSH, catalase, vitamin E); Decrease the production of Aβ	APP/PSEN1 mice	Brain cortex	Monacelli et al. (2017)	
	Vitamin B	Ameliorate cognitive decline by lowering serum homocysteine levels	—	Hippocampus parahippocampal gyrus, inferior parietal lobule and retrosplenial cortex	Douaud et al. (2013); Kennedy, (2016)	
	Deferoxamine	Chelate iron to reduce iron accumulation	APP/PS1 mice	Microglial activation	Feng et al. (2020); Mao et al. (2020); Moreau et al. (2018)	
Other inhibitors	NQO1	Antioxidant stress and lipid peroxidation; Reductase that protects the antioxidant forms of CoQ10, α-tocopherol, and ascorbic acid	—	—	Ross and Siegel, (2021)	
	FSP1	The FSP1-CoQ10-NAD(P)H pathway, together with GPX4 and GSH, inhibits phospholipid peroxidation	HT1080 cells	—	Doll et al. (2019); Chen et al. (2020); Reichert et al. (2020); Stockwell et al. (2020); Yan et al. (2021)
	CoQ10	Inhibit lipid peroxidation	Older mice	Hippocampal striatal cortical function neocortex	Shetty et al. (2013); Yan et al. (2021)
	LA	Blocking tau-induced iron overload, lipid peroxidation and inflammation related to ferroptosis	P301S Tau transgenic mice	Hippocampus and the cortex	Zhang et al. (2018); Song and Long, (2020)

### Aromatic amine antioxidants

Ferrostatin-1 (Fer-1) and liproxstatin-1 (Lip-1), aromatic amine antioxidants, are free radical scavengers that block ROS production and lipid peroxidation.

Fer-1 inhibits ferroptosis much more efficiently than phenolic antioxidants. The anti-ferroptotic activity of Fer-1 is due to the scavenging of initiating alkoxyl radicals produced, with other rearrangement products, by ferrous iron from lipid hydroperoxides. Fer-1 forms a complex with iron, confirmed in cells by calcein fluorescence which indicates decreased labile iron in the presence of Fer-1 (Miotto et al., 2020). In addition, Fer-1 significantly inhibits the production of cytoplasmic and lipid ROS and reverses Glu-induced suppression of GSH and Gpx in HT-22 cells, suggesting that Fer-1 protects HT-22 cells by blocking oxidative toxicity. Therefore, Nrf2 and Gpx4 up-regulation may be the basis of the cytoprotective mechanism of Fer-1 (Chu et al., 2020).

The aromatic amine Lip-1 is the foundation for the antioxidant activity of liproxstatin-1 analogs. It is an excellent radical-trapping antioxidant in phospholipid bilayers, using the bilayers of unilamellar liposomes originating from egg phosphatidylcholine. Lip-1 readily penetrates and remains within the lipid bilayer, permitting its active site to remain in close directional contact with the lipid peroxidation site and initiating the CH_3_OO• extraction of hydrogen atoms from aromatic amine sites (Sheng et al., 2017). Moreover, Lip-1 prevents BODIPY 581/591 C11 oxidation in Gpx4^−/−^ cells but does not interfere with other classical types of cell death, such as TNFα-induced apoptosis and H_2_O_2_-induced necrosis (Friedmann Angeli et al., 2014).

Studies have shown that memory improves in Aβ-induced AD mice when Fer-1 and Lip-1 are administered, and Lip-1 has a more significant effect on memory (Bao et al., 2021). Therefore, aromatic amine antioxidants may offer significant AD treatment options.

### α-Tocopherol

α-Tocopherol, the main type of Vitamin E in tissues, exerts its antioxidant capacity mainly by destroying the chain reaction of automatic oxidation (Zilka et al., 2017). α-Tocopherol transfer protein (TTP) is highly expressed in the brain and regulates the level and distribution of α-tocopherol. Vitamin E and TTP deficiency can lead to oxidative stress in the brain. It has been demonstrated that AD patients have low Vitamin E in the plasma, serum, and cerebrospinal fluid (Ashraf and So, 2020). Moreover, AD patients receiving vitamin E treatment experience slower declines in cognitive function and lower oxidative stress levels than patients receiving the placebo (Boccardi et al., 2016; Gugliandolo et al., 2017; Kryscio et al., 2017).

### Nitroxides

Nitroxides can permeate the cell membranes and cross the blood–brain barrier (BBB). Fe^2+^ is the form of iron found in the LIP *in vivo*. The involvement of the iron (II)-citrate complex in Fenton-like reactions with H_2_O_2_ is considered an *in vivo* mechanism of the LIP that induces oxidative stress and many pathological conditions. The nitroxide Tempo combines with Fe^2+^-citrate to form a Tempo-Fe^2+^-citrate complex, which can effectively inhibit OH production. Nitroxides have significant therapeutic potential as antioxidants in oxidative stress-related diseases (Shi et al., 2017).

### Natural polyphenol compounds

Baicalein is a natural polyphenol compound that inhibits LOXs by reducing oxidative stress and acts as an anti-inflammatory and neuroprotective agent. It inhibits GSH depletion, GPX4 degradation and lipid peroxidation, increases Nrf2, and inhibits 12/15-LOX (Xie et al., 2016; Li et al., 2019; Yuan et al., 2020). Activation of Nrf2 increases iron storage, reduces iron uptake by cells, and limits lipid ROS production (Hassannia et al., 2019; Xu et al., 2021). Baicalein-fed APP/PS1 mice show decreased BACE1 activity, decreased Aβ and p-tau levels, and superior behavioral test results. Another polyphenolic compound, curcumin chelates iron, reduces iron accumulation, scavenges ROS, increases the levels of SOD, Na^+^-K^+^-ATPase, catalase, GSH and mitochondrial complex enzyme (Ege, 2021), inhibits Aβ aggregation, and reduces the effects of P-tau protein. However, curcumin has poor water solubility and has demonstrated inadequate bioavailability in clinical trials. As a result, its application in the clinical treatment of AD is limited (Tang and Taghibiglou, 2017; Reddy et al., 2018; Ege, 2021).

Epigallocatechin gallate (EGCG) is another key polyphenol compound. Found in green tea, it has antioxidant, anti-inflammatory and neuroprotective effects (Plascencia-Villa and Perry, 2021). Treatment of AD mice with EGCG demonstrated that it exerted its protective effects by decreasing the expression of APP and Aβ in the hippocampus. Preclinical studies showed that EGCG has anti-inflammatory and neuroprotective effects against neuron injury and cerebral edema (Cascella et al., 2017).

Decreased melatonin, associated with decreased accumulation of polyphenols, is closely related to AD occurrence. As people age, the pineal gland calcifies, and melatonin secretion gradually decreases (Luo et al., 2020). AD patients experience decreased melatonin synthesis and secretion and abnormal secretion rhythms. Melatonin can reduce oxidative stress and stimulate the synthesis of antioxidant enzymes (SOD, GPX, and glutathione reductase) and GSH production (Balmik and Chinnathambi, 2018). Aβ plasma levels and deposition were found to be significantly reduced in APP/PS1 mice after 12 months of melatonin supplementation. However, clinical trials using melatonin (50–100 mg/day) for 10 days to 24 weeks showed that melatonin is safe but does not improve the cognitive ability of AD patients, only their sleep quality (Plascencia-Villa and Perry, 2021).

The ginkgo biloba tree (*Gingko biloba*) also produces polyphenols with antioxidant effects. After rats were injected with a standardized extract of ginkgo biloba, catalase and superoxide dismutase activities in the hippocampus, striatum, and substantia nigra were increased, lipid peroxidation decreased, and overall oxidative damage was reduced (Plascencia-Villa and Perry, 2021). Finally, the polyphenol derivative CMS121 acts as an antioxidant and inhibits fatty acid synthase to regulate lipid peroxidation levels. CMS121 was shown to improve memory, and cognitive function in APPswe/PS1 δ E9 double transgenic mice (Ates et al., 2020).

### LOX inhibitors

5-Lipoxygenase (5LO) is widely expressed in central nervous system neurons, and its levels increase in an age-dependent manner in the hippocampus and cortex, two brain regions prone to neurodegenerative damage. Studies have shown that 5LO is up-regulated in AD. Zileuton, an anti-inflammatory compound, inhibits LOX5 and decreases γ-secretase, Aβ, and tau after three months of treatment in a 3xTg mouse AD model (Di Meco et al., 2014), demonstrating its broad prospects for clinical use.

### Other inhibitors

APP/PS1 mice treated with the iron chelator deferoxamine demonstrate reduced Aβ and improved memory, but no significant improvement in cognition and memory is observed in AD patients (Feng et al., 2020). Furthermore, patients experience side effects such as loss of appetite and weight. NQO1, a reductase that can maintain antioxidant forms of CoQ10, α-tocopherol and ascorbic acid, plays an important role in maintaining antioxidant protection and inhibiting lipid peroxidation. It has long been associated with the early pathological changes of AD. However, NQO1 increases in the AD brain are limited to brain regions affected by AD pathology. Furthermore, NQO1 production is generally considered a protective response to oxidative stress, which has potential clinical significance in treating AD (Ross and Siegel, 2021).

The inhibition of ferroptosis by ferroptosis suppressor protein 1 (FSP1) is mediated by ubiquinone, also known as coenzyme Q10. U biquinone is converted on the cell membrane into its reduced prototype ubiquinol, which inhibits the peroxide reaction and prevents ferroptosis (Tang et al., 2021). FSP1 catalyzes the regeneration of CoQ10 through NAD(P)H, and the FSP1-CoQ10-NAD(P)H pathway, together with GPX4 and GSH, inhibits phospholipid peroxidation and ferroptosis, offering broad prospects for the treatment of degenerative diseases caused by ferroptosis (Doll et al., 2019; Chen et al., 2020; Mao et al., 2020; Reichert et al., 2020; Stockwell et al., 2020; Yan et al., 2021). GSH is a major antioxidant, combating oxidative stress. One study demonstrated that GSH levels in the hippocampus and cortex are significantly reduced in patients with mild cognitive impairment (MCI) and AD. Although GSH supplementation has been proposed as a therapeutic strategy for MCI and AD, it has not been evaluated in patients in clinical trials (Plascencia-Villa and Perry, 2021).

Vitamin C (ascorbic acid, AA) can increase GSH metabolism and improve cellular oxidative stress. Treatment with high concentrations of AA reduces amyloid plaque formation in the 5XFAD mouse model (Monacelli et al., 2017). Homocysteine is an important intermediate in methionine, folate, and onecarbon metabolism, and elevated homocysteine increases the risk of stroke, age-associated cognitive impairment, and AD. Randomized controlled trials and meta-analyses have indicated that homocysteine-lowering treatments may be recommended to prevent AD. Elevated homocysteine might promote post-stroke cognitive impairment (PSCI) through small vessel disease or AD pathology, which may explain our finding that homocysteine levels are associated with long-term incidence of PSCI (Li et al., 2021). Vitamin B can reduce cognitive decline by lowering serum homocysteine levels. Vitamins B1/B6/B9/B12 were found to improve brain metabolism, oxidative stress, inflammation, and cognition in patients with AD, and folic acid (1.25 mg/day, six months) reduces Aβ and inflammatory biomarkers (TNFα, IL6). Notably, high doses of Vitamin B (folic acid 0.8 mg, Vitamin B6 20 mg, Vitamin B12 0.5 mg) for 2 years was shown to slow the progression of brain atrophy significantly (Donnelly et al., 2008). Another antioxidant, α-lipoic acid, has been found to improve the cognitive function of AD patients by blocking tau-induced iron overload, lipid peroxidation and inflammation related to ferroptosis (Song and Long, 2020).

### Targeted ferroptosis therapy for alzheimer’s disease

The endothelial cells of the BBB are essential in regulating brain iron uptake, and the Tf/TfR1 pathway is the major route for iron absorption in the brain (Yan and Zhang, 2019). However, the BBB presents challenges for the passage of some drug therapies into the brain. Therefore, developing effective nanomaterial carriers is crucial to improving drug delivery, release, and targeting efficiency. Nanomaterials that deliver drugs targeting ferroptosis have been extensively examined in recent years. Studies have shown that transferrin nanomaterials can penetrate the BBB and deliver drug molecules to the central nervous system (Luo et al., 2021; Zheng et al., 2021), thus providing new AD therapeutic options.

GSH levels decrease with age and possible development of AD. Few studies have focused on exploring the role of exosomes in the metastasis of GSH or its precursors to enhance and supplement intracellular GSH, especially in neuronal cells. More research is needed to understand the potential role of exosomes in oxidative stress and neuroprotection, including GSH transfer. GSH may also be used as a targeted ligand for translocation across the BBB *via* nanocarriers to treat various brain dysfunctions (Haddad et al., 2021). Moreover, therapy targeted to GSH degradation can effectively treat ferroptosis-mediated organ injury (Jiang et al., 2020).

Autophagosome accumulation is a significant feature in human AD patients and animal model neurons. Increased production and accumulation of Aβ in lysosomes has been observed in autophagy-deficient cells, suggesting that the turnover portion of Aβ is regulated by autophagy. As autophagy-related genes are highly expressed in early AD, enhanced autophagy may be a promising research area for achieving neuroprotection in AD patients (Moujalled et al., 2021).

Nrf2 target genes have been shown to be involved in GPX4 synthesis and function, intracellular iron homeostasis, and lipid peroxidation clearance. The Nrf2 protein regulates GSH and thioredoxin-based antioxidant systems (e.g.,TXN1, TXNRD1). Targeting the antioxidant transcription factor Nrf2 to inhibit ferroptosis is a promising new option for neurodegenerative control and significant in the study of human nervous system diseases and aging, especially neurodegenerative diseases such as Parkinson’s disease, AD, and Huntington’s disease (Song and Long, 2020).

The tau protein plays an important role in stabilizing microtubules. In AD pathology, if the protein is over-phosphorylated, it will separate from microtubules, leading to axonal microtubule disintegration (Wang and Mandelkow, 2016). Where microtubules are destroyed, and tau oligomers (tauO) are pathological, p53 cannot enter the nucleus. Over time, p53 outside the nucleus may become unstable and start to aggregate, and tauO near the nucleus interacts with p53 to form a p53 oligomer. Cell cycle arrest, DNA damage repair, apoptosis, and other crucial functions can be compromised when p53 is not allowed to enter the nucleus. Since the cell cannot be repaired and cell death cannot be controlled, conditions inside the cell will continue to deteriorate, promoting the accumulation of other disordered proteins. Targeting pathological tau proteins, especially tauO, may prevent p53 aggregation and destruction (Farmer et al., 2020). Since p53 controls many cellular functions, affecting this key transcription factor may lead to irreversible AD pathology. A deeper understanding of p53’s role in AD lesions is therefore warranted.

## Conclusion

Compared with other tissues and organs of the human body, brain tissue is rich in PUFA and iron. It consumes substantial oxygen, so it is prone to lipid peroxidation, poor antioxidant capacity, and higher ferroptosis sensitivity. The occurrence of ferroptosis is closely related to the regulation of iron homeostasis, the ferroptosis signal pathway, and amino acid metabolism. AGEs are highly detectable in the blood and cerebrospinal fluid of patients with neurodegenerative diseases such as AD (Bär et al., 2003). As an important part of the ferroptosis antioxidant system, GSH helps eliminate AGEs, so increasing the level of GSH in the brain is a new strategy for treating AD. GCL is the rate-limiting enzyme of GSH synthesis, and when its activity decreases, GSH content can be reduced and GPX4 inactivated, leading to the accumulation of lipid peroxidation. This accumulation will further increase ROS and ultimately lead to ferroptosis. Therefore, the effective synthesis of GSH is a new direction for AD treatment.Mitochondria are the main source of ROS. When antioxidant factors are unbalanced within mitochondria, oxidative stress will lead to the release of Glu in neurons. High extracellular Glu will inhibit system xc^−^, resulting in ferroptosis. Moreover, Glu transporter expression in the cerebral cortex of AD patients and VGLUT1 significantly decreased, possibly resulting in poor clearance of glutamic acid in the synaptic cleft, leading to excitatory toxicity. Research on targeted therapy to reduce excitatory toxicity of Glu is promising for future AD treatment.

The mechanism of AD-mediated ferroptosis is gradually being clarified, and iron inhibitors have permitted some progress in AD treatment. This progress notwithstanding, the regulatory factors that regulate the ferroptosis signaling pathway vary and are dependent upon the AD stage. Therefore, the inducing factors and specific mechanisms of ferroptosis in AD remain to be elucidated and should be the focus of next studies. AD is a complex and multifactorial chronic disease. The clinical benefits of ferroptosis inhibitors in AD and their effects on other tissues are the focus of much current research. Whether a single drug or intervention targeting iron death can avoid, reduce or reverse AD requires substantial analysis. In-depth research on the different stages of AD involved in ferroptosis will help us develop a more comprehensive understanding of the AD onset and progression mechanisms and provide a more rigorous theoretical basis for prevention and treatment.
